# Personalized Medicine—Current and Emerging Predictive and Prognostic Biomarkers in Colorectal Cancer

**DOI:** 10.3390/cancers12040812

**Published:** 2020-03-28

**Authors:** Christine Koulis, Raymond Yap, Rebekah Engel, Thierry Jardé, Simon Wilkins, Gemma Solon, Jeremy D. Shapiro, Helen Abud, Paul McMurrick

**Affiliations:** 1 Cabrini Monash University Department of Surgery, Cabrini Health, Malvern 3144, VIC, Australia; rayyap@gmail.com (R.Y.); rengel@cabrini.com.au (R.E.); SimonWilkins@cabrini.com.au (S.W.); gemmasolon@gmail.com (G.S.); pjm@colorectal.com.au (P.M.); 2 Department of Anatomy and Developmental Biology, Monash University, Clayton 3800, VIC, Australia; thierry.jarde@monash.edu (T.J.); helen.abud@monash.edu (H.A.); 3 Monash Biomedicine Discovery Institute, Stem Cells and Development Program, Monash University, Clayton 3800, VIC, Australia; 4 Centre for Cancer Research, Hudson Institute of Medical Research, Clayton 3168, VIC, Australia; 5 Department of Epidemiology and Preventive Medicine, Monash University, Melbourne 3000, VIC, Australia; 6 Cabrini Haematology and Oncology Centre, Cabrini Health, Malvern 3144, VIC, Australia; jeremy.shapiro@monash.edu

**Keywords:** biomarkers, colorectal cancer, predictive, prognostic, organoid, consensus molecular subtypes

## Abstract

Colorectal cancer (CRC) is the third most common cancer diagnosed worldwide and is heterogeneous both morphologically and molecularly. In an era of personalized medicine, the greatest challenge is to predict individual response to therapy and distinguish patients likely to be cured with surgical resection of tumors and systemic therapy from those resistant or non-responsive to treatment. Patients would avoid futile treatments, including clinical trial regimes and ultimately this would prevent under- and over-treatment and reduce unnecessary adverse side effects. In this review, the potential of specific biomarkers will be explored to address two key questions—1) Can the prognosis of patients that will fare well or poorly be determined beyond currently recognized prognostic indicators? and 2) Can an individual patient’s response to therapy be predicted and those who will most likely benefit from treatment/s be identified? Identifying and validating key prognostic and predictive biomarkers and an understanding of the underlying mechanisms of drug resistance and toxicity in CRC are important steps in order to personalize treatment. This review addresses recent data on biological prognostic and predictive biomarkers in CRC. In addition, patient cohorts most likely to benefit from currently available systemic treatments and/or targeted therapies are discussed in this review.

## 1. Introduction

Colorectal cancer (CRC) represents the third most common cancer in developed countries and is a leading cause of cancer deaths worldwide [1], highlighting the need to study predictive markers for response to available and emerging therapies. Treatment for CRC is largely determined by the pathologically based tumor/node/metastasis (TNM) staging system. The evolving understanding of the genetic heterogeneity of CRC suggests however, that a purely pathologic classification is insufficient to determine optimal therapy. The model of progressive stepwise accumulation of genetic and epigenetic events leading to adenoma and carcinoma formation is well described [2]. This includes ‘driver’ alterations in tumor suppressor genes and oncogenes, leading to the currently utilized predictive and prognostic clinical biomarkers such as microsatellite instability (MSI) due to deficient mismatch repair (dMMR), Kirsten rat sarcoma viral oncogene homolog (*KRAS*), v-raf murine sarcoma viral oncogene homolog B (*BRAF*) and mutational status of various single genes (e.g., *KRAS* and *BRAF*).

In this review, we discuss current prognostic and predictive clinical biomarkers, including those that guide therapy and those associated with familial cancers (summarized in Table 1). The advent of new technologies characterizing the molecular mechanisms underlying tumorigenesis has resulted in the emergence of many potential new biomarkers, including consensus molecular subtypes (CMS), stem cell markers, circulating tumor DNA (ctDNA), cell-free DNA (cfDNA), genetic alterations, immune- and apoptosis-related biomarkers, which will be outlined in this review (summarized in Table 2).

## 2. An Overview of CRC Classification and Molecular Pathways

CRC is a heterogeneous disease that can be currently classified according to its global genomic status in terms of MSI and chromosomal instability (CIN) and epigenomic status as expressed by CpG island methylator phenotype (CIMP). These molecular genetic and epigenetic changes act to dysregulate conserved signaling pathways resulting in the transformation of normal colonic epithelium to an intermediate adenoma and ultimately to an adenocarcinoma.

The CIN pathway is responsible for approximately 65–70% of sporadic CRC [113] and is characterized by an imbalance in chromosome number (aneuploidy), chromosomal genomic amplifications and a high frequency of loss of heterozygosity (LOH), commonly occurring through mutations in *APC* and *KRAS* [114]. A small proportion of CIN tumors are inherited and arise secondary to germline mutations in the *APC* gene as seen in familial adenomatous polyposis (FAP) or the *MUTYH* gene (as seen in MUTYH-associated polyposis) [115].

The MSI pathway occurs in 15% of CRC and can be sporadic. This pathway is characterized by dMMR proteins resulting in insertion and deletion mutations in stretches of short tandem DNA repeats (microsatellites) as well as nucleotide substitutions throughout the genome. The detection of instability is identified via a PCR-based assay categorizing tumors as either MSI-high (MSI-H), MSI-low (MSI-L) or microsatellite stable (MSS), based on the number of microsatellite markers demonstrating instability [116].

The CIMP pathway is characterized by epigenetic alterations, resulting in changes in gene expression or function without changing the DNA sequence of that particular gene. These epigenetic changes are usually caused by DNA methylation or histone modifications. DNA methylation occurs commonly at the 5′-CG-3′ (CpG) dinucleotide. Methylation of gene promoter region results in gene silencing, thus providing an alternative mechanism for loss of function of tumor suppressor genes. Genes involved in CRC that are silenced by DNA hypermethylation include *APC* and *MLH1* [113]. Testing for CIMP is performed via PCR for hypermethylation in *CACNA1G*, *MLH1*, *NEUROG1*, *RUNX3* and *SOCS1* [37].

The classification of CRC consensus molecular subtypes (CMS) was formed in an effort to understand the heterogeneous clinical and drug outcomes observed in CRC patients, even when controlled for similar pre-operative prognostic features, tumor stage and clinicopathological characteristics [117,118,119,120,121,122]. Each CMS has distinguishing expression data and pathways and are designated CMS1 (microsatellite instability immune), CMS2 (canonical), CMS3 (metabolic), CMS4 (mesenchymal) and a mixed features phenotype representing transitional or intratumoral heterogeneity [123]. CMS can be determined through gene expression analysis, however, recently five immunohistochemistry-based classifiers, CDX2, FRMD6, HTR2B, ZEB1 and KER have been identified that demonstrate 87% concordance with traditional transcriptome-based classification [124]. The recent classification of four CMS may form the basis for future clinical stratification of CRC with subtype-based targeted interventions.

## 3. An Overview of Current CRC Therapeutics

The current medical treatment for CRC involves a mix of surgery, chemotherapy protocols and the inclusion of monoclonal antibody therapy [125]. Selected treatment options are now dependent on a range of factors including stage, patients’ health status, initial treatment intent (curative vs. palliative), clinical features such as tumor location and molecular factors (e.g., *RAS*, *BRAF* mutational status). These factors play important prognostic roles and may also predict a patient’s response to treatment.

Fluorouracil (5-FU; an anti-metabolite fluoropyrimidine agent) continues to be the most widely used agent for CRC and provides modest improvements in progression-free survival (PFS), disease-free survival (DFS), overall survival (OS) and response rate (RR) both in the adjuvant setting and in metastatic CRC (mCRC) [126]. Oxaliplatin and irinotecan (anti-neoplastic agents) and antibodies targeting vascular endothelial growth factor (VEGF) and epidermal growth factor receptor (EGFR), have provided incremental gains in RR, PFS and OS in advanced disease, with only oxaliplatin enhancing fluoropyrimidine-based adjuvant chemotherapy.

## 4. Current Prognostic and Predictive Biomarkers

Clinical biomarkers can be prognostic, predictive or both. Prognostic biomarkers are an independent measure of the course of a disease in an untreated population. The presence or absence of a biomarker is associated with a patient’s overall clinical outcome (i.e., risk of recurrence and mortality). Prognostic biomarkers in CRC provide treatment-independent prognostic information on patient outcomes and include dMMR/MSI, *KRAS*, *BRAF* and CEA. Conversely, predictive biomarkers help to assess whether a biomarker-positive individual will respond beneficially to a specific therapeutic intervention. In CRC, predictive biomarkers for toxicity to irinotecan and 5-FU include, *UGT1A1* and dihydropyrimidine dehydrogenase (DPD) deficiency, respectively. Lastly, a biomarker that confers both prognostic and predictive qualities in CRC is primary tumor location; right-sided CRC is associated with a poorer prognosis than left-sided CRC [127] and left-sided CRC predicts response to EGFR-targeted therapies [12]. Current prognostic and predictive biomarkers are discussed in this section and summarized in Table 1.

### 4.1. Biomarkers that Guide Therapy

#### 4.1.1. Mismatch Repair Deficiency (dMMR)

DNA damage repair proteins exist to facilitate the replication of normal cellular DNA [3]. The MSI phenotype results when this protective mechanism is lost through dMMR. Critical mismatch repair (MMR) proteins involved in proofreading and correction of insertion-deletion loops include MLH1, MSH2, MSH6 and PMS2. Deficiencies in MMR can occur through either a germline mutation in an MMR gene (*MLH1*, *MSH2*, *MSH6* and *PMS2*), resulting in Hereditary Nonpolyposis Colorectal Cancer (HNPCC) also known as Lynch syndrome, however, this condition occurs more commonly through sporadic epigenetic inactivation of *MLH1* [3]. The latter is generally associated with hypermethylation of promoter regions of cancer-specific genes known as the CIMP-H.

Currently, the routine use of adjuvant chemotherapy is not recommended in stage II CRC patients. Exceptions to this include those at higher risk of recurrence, for example tumors with adverse features such as poor differentiation, lymphovascular or perineural invasion and younger age patients. dMMR provides a molecular, tailored approach to stratifying patients based on their potential response to chemotherapy. Sargent and colleagues described dMMR as a predictive biomarker for poor response to FU-based adjuvant therapy in stage II and stage III colon cancer [4]. This is highlighted in their study of 457 colon cancer patients who were randomly assigned to either FU-based therapy or no post-surgical treatment. Patients with dMMR tumors receiving FU-based therapy had no improvements in DFS (hazard ratio [HR], 1.10; 95% CI, 0.42 to 2.91; *p* = 0.85), when compared to those assigned to surgery alone. In contrast, patients with microsatellite-stable or proficient MMR (pMMR) tumors, receiving adjuvant therapy, demonstrated significantly improved DFS (HR, 0.67; 95% CI, 0.48 to 0.93; *p* = 0.02) [4]. Similarly, in an Australian cohort study, patients with dMMR, despite not being given adjuvant chemotherapy, still had excellent outcomes [5]. These studies support the concept that adjuvant chemotherapy in stage II colon cancer patients with high dMMR results in minimal OS benefit (2–3%) and as such it is not routinely recommended [4,5,6].

MSI high tumors are associated with a better prognosis in curative settings, however, in mCRC, it appears to confer a negative prognosis. As a predictive biomarker, a large amount of evidence suggests possible resistance to 5-FU in MSI-H tumors [10]. This is due to the high mutational load eliciting an endogenous immune anti-tumor response, which is counterbalanced by the expression of immune inhibitory signals, such as PD-1 or PD-L1, resisting tumor elimination [11]. Based on these considerations, MSI-H CRCs are highly responsive to immunotherapy, such as anti-PD-1 [11]. Current guidelines recommend MSI testing in all CRC patients, to not only identify HNPCC but to guide adjuvant treatment decisions and to identify patients likely to benefit from immunotherapy in stage IV disease.

HNPCC is an autosomal dominant condition caused by genomic mutations in DNA MMR genes, *MLH1*, *MSH2*, *MSH6* and *PMS2* [7]. Inactivating mutations in the MMR result in a high level of MSI (MSI-H) and subsequently an increased risk of cancer, particularly colon and endometrial [8].

#### 4.1.2. KRAS

*KRAS* belongs to the *RAS* family of oncogenes and is mutated in 40–50% of CRCs [13], most commonly via point mutations [14,15]. Whilst some studies have suggested a prognostic role for *RAS* [16,17,18,19], its main utility is as a predictive biomarker. Tumors with a mutation in codon 12 or 13 of exon 2 of the *KRAS* gene are essentially unresponsive to EGFR-antibody (EGFR-ab) therapy. Similarly, mutation in *KRAS* outside of exon 2 and mutation in *NRAS* are predictive for poor response to EGFR-ab therapy [20,21]. This is also highlighted in studies by Karapetis et al. and Amado et al., who demonstrated that *KRAS* predicts response to cetuximab and panitumumab in advanced CRC, respectively [68,69]. The study by Karapetis et al., correlated tumor mutation status of the *KRAS* gene with survival in advanced CRC patients receiving either cetuximab or supportive care. Their study found that for patients with wild-type *KRAS* tumors (*KRAS^WT^*), treatment with cetuximab as compared with supportive care alone significantly improved OS (median, 9.5 vs. 4.8 months; HR for death, 0.55; 95% CI, 0.41 to 0.74; *p* < 0.001) and PFS (median, 3.7 months vs. 1.9 months; HR for progression or death, 0.40; 95% CI, 0.30 to 0.54; *p* < 0.001) [68]. Similarly, Amado and colleagues assessed the impact of *KRAS* mutations on PFS in mCRC patients on PFS following treatment with panitumumab. They found PFS was significantly greater in patients receiving panitumumab with *KRAS^WT^*, (HR, 0.45; 95% CI: 0.34 to 0.59) than in the mutant group (HR, 0.99; 95% CI, 0.73 to 1.36) [69]. These practice-changing discoveries have defined restrictions for the use for EGFR-ab therapy to patients with mCRC with wild-type *RAS* (*RAS^WT^*), sparing up to 60% of patients’ futile exposure to toxicity and saving needless cost [22].

An additional consideration in *RAS^WT^* patients is the impact of tumor sidedness on targeted therapy. A recent study by Holch and colleagues investigated the prognostic and predictive relevance of primary tumor location. Their meta-analysis of first line clinical trials concluded that patients with left-sided *RAS^WT^* mCRC had significantly greater survival benefit from anti-EGFR treatment compared with anti-VEGF treatment when added to standard chemotherapy (HR, 0.71; 95% CI, 0.58–0.85; *p* = 0.0003). In contrast, patients with right-sided *RAS^WT^* mCRC demonstrated significantly improved PFS when treated with chemotherapy plus VEGF-ab therapy (HR, 1.53; 95% CI, 1.16–2.01; *p* = 0.003) [12]. Nonetheless, due to the molecular heterogeneity within left- and right-sided tumors, caution regarding treatment decisions needs to be exercised when basing therapy on the location of tumor in the colon [123,128].

#### 4.1.3. BRAF (V600E)

The prognostic impact of the most common *BRAF* mutation in mCRC, *BRAF^V600E^*, is well characterized [129]. The survival rate of patients carrying the mutant form is decreased by approximately 50% compared to patients with wild-type *BRAF* (*BRAF^WT^*) [130,131,132,133]. This is highlighted in a pooled analysis of the CAIRO, CAIRO2, COIN and Focus studies, where patients with *BRAF* mutations demonstrated worse median PFS and OS compared with patients that had *BRAF^WT^* tumors (PFS: 6.2 vs. 7.7 months, respectively; HR, 1.34; 95% CI, 1.17–1.54; *p* < 0.001; OS: 11.4 vs. 17.2 months, respectively; HR, 1.91; 95% CI, 1.66–2.19; *p* < 0.001) [133]. However, it is important to note that not all *BRAF* mutations exhibit the same clinical behavior. One previous study has suggested that *BRAF^non-V600E^* mutations have more favorable outcomes compared to *BRAF^V600E^* mutation or *BRAF^WT^* tumors in mCRC (60.7 vs 11.4 vs. 43.0 months, respectively; *p* < 0.001) [23]. In addition to prognostic implications, *BRAF* mutations may serve as a predictive biomarker for triplet combination therapy of mitogen-activated protein kinase (MEK), *BRAF* inhibition plus EGFR-targeted therapies. This is highlighted in a recent phase II study by Corcoran and colleagues who found patients with *BRAF^V600E^* mCRC receiving triplet therapy had a 21% response rate (95% CI, 12.5–43.3%) compared to 10% response for patients in the dabrafenib plus panitumumab arm (95% CI, 1.2–31.7%) [134]. In addition, the ongoing phase III BEACON CRC trial, where patients are receiving triplet combination of binimetinib, encorafenib and cetuximab, demonstrated an overall response rate of 48% (95% CI, 29.4% to 67.5%), median PFS of 8 months (95% CI, 5.6 to 9.3 months) and median OS of 15.3 months (95% CI, 9.6 months to not reached) [135]. Based on these results, the US Food and Drug Administration (FDA) granted a Breakthrough Designation to this triplet therapy for *BRAF^V600E^* CRC patients whom failed one or two prior lines of therapy for metastatic disease [136].

Somatic *BRAF* mutations, most frequently V600E, have been described in a significant proportion of sporadic MSI-H CRC but not in HNPCC. Thus, clinical *BRAF* mutation testing has been proposed as a means to identify MSI-H CRC cases that do not require germline MMR gene testing [24].

#### 4.1.4. Carcinoembryonic Antigen (CEA)

CEA is one of the most extensively used tumor markers worldwide [25]. Despite its poor sensitivity and specificity [26,27], a rising CEA post curative surgery often correlates with relapse. Thus, CEA is useful in providing early detection of recurrence and allows clinicians a means for early detection and surgical resection of metastases [25,28]. The benefits of CEA as a reliable predictor or recurrence and survival after curative surgery in patients with CRC is highlighted in a recent retrospective study by Baqar and colleagues. In their study of 623 CRC patients, elevated CEA (≥5 ng/mL) was a predictor of recurrence (HR, 1.8; 95% CI, 1.09–3.00; *p* = 0.002) and of OS (HR, 7.79; 95% CI, 1.00–3.19; *p* = 0.046) [28].

#### 4.1.5. Irinotecan Toxicity and UGT1A1*28

Irinotecan hydrochloride is an anti-neoplastic topoisomerase inhibitor that is widely used in combination with 5-FU and leucovorin chemotherapy for first line treatment of mCRC and as a single agent in second-line salvage therapy of 5-FU refractory mCRC. The principle dose-limiting toxicities associated with irinotecan are delayed diarrhea and severe neutropenia; these toxicities are reversible, not cumulative and related to irinotecan dose [137]. Irinotecan is metabolized into toxic 7-ethyl-10-hydroxycamptothecin (SN-38) via the hepatic enzyme uridine diphosphate-glucuronosyltransferase 1A (UGT1A) and the inactivated byproduct, SN-38, excreted in bile. The effect of genetic polymorphisms of the *UGT1A1* gene in predicting irinotecan-associated toxicity has gained interest. Currently, over 100 genetic variants of *UGT1A1* exist, the wild-type allele, UGT1A1*1, being associated with normal enzyme activity and the most common variant allele, UGT1A1*28, being investigated as a cause for increased irinotecan toxicity [138]. The findings from four pharmacogenetic trials, assessing the impact of several *UGT1A1* variants, found that patients homozygous for UGT1A1*28 experienced significantly more serious hematological side effects [139,140,141,142]. Based on this evidence, the United States (US) Food and Drug Administration (FDA) amended the irinotecan label in 2005 to include UGT1A1*28/*28 as a risk factor for severe neutropenia, stating that when administered as a single-agent, a reduction in the starting dose by at least one level or irinotecan hydrochloride injection should be considered for patients known to be homozygous for the UGT1A1*28 allele [138]. In addition, Hoskins and colleagues performed a meta-analysis assessing the association of irinotecan dose with the risk of irinotecan-related hematologic toxicities (grade III-IV) based on *UGT1A1* variants. Their findings concluded that the risk of toxicity was higher among patients with UGT1A1*28/*28 genotype than among those with UGT1A1*1/*1 or UGT1A1*1/*28 genotype for both medium (OR = 3.22; 95% CI, 1.52 to 6.81; *p* = 0.008) and high (OR = 27.8; 95% CI, 4.0 to 195; *p* = 0.005) doses of irinotecan, only. Despite black box warnings in the US by the FDA, these warnings have not been replicated in other jurisdictions such as Australia most likely due to conflicting studies [143,144,145]. In summary, despite the significance of UGT1A1*28 as a potential biomarker for irinotecan toxicity, genotyping for *UGT1A1* is not current clinical practice for determining risk of hematologic toxic effects. Instead, the current clinical protocol suggests close clinical monitoring for patients receiving irinotecan, particularly during the first cycle of chemotherapy, with drug doses adjusted based on standard clinical tests such as white blood cell counts.

#### 4.1.6. 5-FU Toxicity and DPD Deficiency

The *DPYD* gene encodes the enzyme dihydropyrimidine dehydrogenase (DPD), which functions as the rate-limiting step in the metabolism of fluoropyrimidine chemotherapies [146]. Greater than 80% of 5-FU is metabolized by DPD, with factors such as age, race, comorbidities and concomitant therapies influencing metabolism. Reduced activity of DPD impacts on the ability to metabolize 5-FU at normal rates and may result in life threatening toxicity [30]. *DPYD* variants that do not affect DPD activity in a clinically relevant manner include c.85T > C, *9A, rs1801265, p.C29R; c.1627A > G, *5, rs1801159, p.I543V; c.2194G > A, *6, rs1801160, p.V7321. Conversely, variants that have been shown to have deleterious effects on DPD activity, resulting in 5-FU toxicity, include DYPD*2A and DPYD*13. While variants c.2846A > T and c.1129–5923C > G have been shown to have moderately reduced DPD activity [147]. A multicenter study of 17 hospitals assessing *DPYD* genotype-guided dosing in patients receiving fluoropyrimidines (capecitabine or fluorouracil) was carried out by Henricks and colleagues [148]. Their study found *DPYD* genotype-based dose reductions improved patient safety and fluoropyrimidine treatment. Specifically, patients with either the DPYD*2A or c.1679T > G variant benefited from an initial 50% dose reduction of fluoropyrimidines. While patients that were c.1236G > A or c.2846A > T carriers, a 25% dose reduction was not enough to lower the risk of severe toxicity and a larger dose reduction of 50% was suggested in these patients. The authors highlight the need for additional prospective studies to validate and further refine these findings [148]. Currently prospective testing for *DPYD* mutations is not routinely performed in clinical practice due to associated costs (approximately $300 in Australia) and long test turnaround times (3–4 weeks) which can be unsatisfactory for developing a therapeutic strategy for patients who require immediate treatment. Thus, for *DPYD* testing to be routinely used in clinical practice, the problematic turnaround time and lack of funding for tests are barriers that would need to be overcome.

### 4.2. Biomarkers Associated with Familial Cancer Syndromes

#### 4.2.1. Adenomatous Polyposis Coli (APC)

*APC* is a tumor suppressor gene that is mutated in more than 80% of CRCs and is a common germline mutation in the autosomal dominant FAP syndrome [31]. This disease is characterized by numerous colonic adenomas which, without recognition and intervention, results in the development of early-onset CRC [32]. Clinical diagnosis of FAP is based on genetic testing of the *APC* gene via an in vitro synthesized-protein assay [33]. A positive test justifies surveillance and familial screening with colorectal endoscopy and aids in surgical management and planning.

#### 4.2.2. MLH1, MSH2, MSH6 and PMS2

As previously discussed in Section 4.1.1, diagnosis for HNPCC involves confirmation of a pathogenic germline mutation in one of several DNA MMR genes, including *MLH1*, *MSH2*, *MSH6* and *PMS2* and/or loss of DNA MMR proteins via immunohistochemistry (IHC) [9]. Germline testing is usually performed on patients with MSI as identified by IHC and in whom acquired methylation has been excluded.

#### 4.2.3. SMAD4 and BMPR1A

Juvenile polyposis syndrome (JPS) is an autosomal dominant disorder characterized by the occurrence of juvenile polyps predominantly in the gastrointestinal tract, resulting in an increased risk of CRC [149]. Genetic testing for germline mutations of *SMAD4* or *BMPR1A* genes are found in approximately 40–60% of JPS cases [150].

## 5. Emerging Prognostic and Predictive Biomarkers

### 5.1. Consensus Molecular Subtypes

The recent classification of four CMS may form the basis for future subtype-based targeted interventions in CRC patients. A growing number of exploratory studies are uncovering CMS-dependent prognostic factors with a potential role for CMS-based therapeutic strategies. This research is more prevalent in mCRC, with recent research highlighting CMS could be potentially used as a predictive biomarker for the efficacy of chemotherapeutic agent regimens. A study by Okita and colleagues showed that irinotecan is highly effective in CMS4 patients [35]. In addition, Mooi and colleagues show that patients with CMS2 and possibly CMS3 preferentially benefit from the addition of bevacizumab to first line capecitabine-based chemotherapy in mCRC compared with other CMS groups [36] (Table 2). Although promising, the association of CMS with treatment and survival outcomes requires further validation through larger retrospective and prospective studies. For CMS to be utilized and feasible in a clinical setting, obstacles such as improved standardization and reproducibility of transcriptomic, genomic and proteomic approaches will need to be overcome.

### 5.2. CpG Island Methylator Phenotype (CIMP)

As discussed in Section 2, tumors occurring through the CIMP pathway that display hypermethylation in the promoter regions of the tumor-suppressor gene or other tumor-related genes, are referred to as CIMP-H or CIMP-L [37,38]. CIMP-H colorectal carcinomas have a distinct clinical, pathologic and molecular profile, including associations with female gender, proximal tumor location, mucinous-type and poor differentiation, dMMR and *BRAF* mutations [39]. Currently, CIMP is one of the most widely used features for sorting subgroups of CRC for the purpose of biomarker and therapeutic research strategy development [40]. The prognostic value of CIMP has been explored in two retrospective studies and a post-hoc analysis of the CALGB 89803 prospective trial [39,151,152]. These three studies suggest that CIMP-H tumors have worse survival compared to CIMP tumors. However, it is important to note that due to the overlap of CIMP with *BRAF* mutations and MMR status, further studies are warranted. An example of this is observed in CIMP-H tumors found to be MSI-positive, harboring the *BRAF* mutation results in a good prognosis [41]. However, MSI-negative tumors which are positive for CIMP and the *BRAF* mutation have a poor prognosis [153] (Table 2). Thus, the independent value of CIMP needs to be validated before being clinically valuable.

### 5.3. DNA Aneuploidy

DNA aneuploidy, an accepted marker for CIN, is found in the majority of sporadic CRCs and has been linked to poor prognosis [44,45]. This is supported by two recent meta-analyses which demonstrated poorer OS for patients with stage II–III cancer exhibiting CIN [46,47]. In addition, CIN has been shown to act as an independent predictor of early relapse and death in stage II patients [48]. These studies suggest that DNA aneuploidy may be a potential predictive biomarker, however, further validation of the prognostic value of DNA aneuploidy is required (Table 2).

### 5.4. Stem Cell Markers

The use of cancer stem cell (CSC) markers as prognostic biomarkers and therapeutic targets is promising and innovative. The CSC hypothesis supports a model where a small population of stem cells drive tumor growth, metastasis and may even predict disease relapse [49] (Table 2). Furthermore, CSC may enter a quiescent state, rendering them inherently resistant to anti-proliferative drugs and subsequently driving tumor recurrence following therapy. There is growing interest in a number of colorectal CSC markers such as CD44, BMI-1, LgR5, EphB2, CD24, CD29, CD133, CD144, CD166 and CXCR4, however, conclusive experimental evidence for their functional relevance is still lacking. Here, we highlight in further detail four of the above CSC markers that demonstrate potential clinical roles for predicting cancer recurrence, therapy resistance and prognosis in CRC.

CD44 is a transmembrane glycoprotein that regulates cell-cell interactions, cell adhesion and migration [154]. Variant isoforms of CD44, created by alternate splicing of the mRNA, are associated with stem cell potential and cancer progression, with the expression of the CD44v6 isoform in colon cancer identified as an independent negative prognostic marker [50,51,52,53,54,155]. CD44v6 is required for tumor migration and by engaging with hepatocyte growth factor receptor, it activates an epithelial-mesenchymal transition program promoting cell motility and invasiveness, thus exhibiting potential as both a functional prognostic biomarker and a therapeutic target in CRC [53].

Expression of oncogene B cell specific moloney leukemia virus integration site-1 (BMI-1) has been shown to be upregulated in CRC tissue compared to corresponding normal tissue [156,157,158]. Involved in the regulation of stem cell renewal [159,160], BMI-1 fosters malignant transformation in CRC [161]. It has shown some promise as independent prognostic factor for OS and DFS in colon cancer, with high expression of BMI-1 associated with poor outcomes [55,157,162]. Additionally, a recent study by Tsai et al., (2019), reveals that expression of BMI-1 is associated with efficacy of chemotherapeutic drug, paclitaxel, suggesting that treatment with this drug should be specifically indicated for CRC patients with low BMI-1 expression [163].

Normal intestinal stem cell markers, including Lgr5 and EphB2, have been shown to be over-expressed in CRC tissue compared to normal colonic mucosa [56]. Importantly, the expression of a stem cell signature, that included Lgr5 and EphB2, was associated with more aggressive colorectal tumors and predicted disease relapse [164]. Genetic ablation of Lgr5+ stem cells in colorectal tumors of transgenic mice indicated a critical role for Lgr5+ cells in the establishment and maintenance of CRC-derived liver metastasis [57]. However, it was recently demonstrated that most circulating CRC stem cells are in fact Lgr5- cells, capable of seeding CRC metastatic lesions in which Lgr5+ cells were present [165]. The ability of differentiated cancer cells to form metastases and re-establish the cellular hierarchy highlights the need to target endogenous cellular plasticity in order to inactivate metastatic potential. More recently, following the identification of a novel stem cell population it was shown that a marker of the revival stem cell population, Clusterin, may predict resistance to 5-FU based chemotherapies; however, these preliminary observations require further validation [58]. Targeting the CSC population in mCRC represents a powerful strategy for future treatments, however, a robust biomarker that can be utilized in the clinic is yet to be developed.

### 5.5. Circulating Tumor DNA (ctDNA) and Cell-Free DNA (cfDNA)

ctDNA and cfDNA offer a promising non-invasive alternative for real-time monitoring of tumor evolution and therapeutic response compared to traditional tissue biopsy. Firstly, ctDNA is emerging as a potentially promising biomarker for the detection of tumor-specific DNA mutations in the cell-free component of peripheral blood with the fraction of ctDNA ranging from 0.01% to 90% [166,167,168]. The possibility that ctDNA could be used to detect micrometastatic disease in patients undergoing surgery with curative intent was first trialed in an initial series of 18 patients with advanced CRC undergoing metastasectomy [59] and subsequently in the setting of breast and pancreatic cancers [60,61]. In stage II CRC, ctDNA analysis may be used as an indicator of minimal residual disease, allowing for identification of patients who would eventually develop recurrent disease [62] (Table 2). This is of immense prognostic value and is subsequently being evaluated via the current prospective DYNAMIC trial which aims to determine the effect of ctDNA in guiding adjuvant chemotherapy use on recurrence-free survival in stage II CRC [62,63].

cfDNA characterizes DNA freely circulating in the bloodstream, not necessarily of tumor origin and is released through apoptosis and necrosis [169]. In cancer, an increase in cellular turnover results in higher levels of cfDNA. Observational studies have reported that the half-life of cfDNA in circulation varies from several minutes to 1–2 h [170], allowing cfDNA to provide a “real-time” snapshot of disease burden. Basnet and colleagues performed a meta-analysis to understand the prognostic significance cfDNA in CRC [64]. Their study showed that detection of cfDNA in plasma was associated with an inferior recurrence-free survival (HR, 2.78; 95% CI, 2.08–3.72) and OS (HR, 3.03; 95% CI, 2.51–3.66) in CRC patients, irrespective of disease stage, study size, tumor markers, detection methods and marker origin [64]. Similarly, El Messaoudi and colleagues assessed cfDNA in 97 mCRC patients and found high levels of cfDNA in plasma was associated with significantly shorter OS (18 months vs. 28.5 months; *p* = 0.0087) [65].

There are a large number of studies that have correlated cfDNA with *BRAF* and *KRAS* tissue mutations [168,171,172,173]. In a prospective-multicenter study, Thierry and colleagues show that cfDNA analysis could advantageously replace tumor-section analysis for *KRAS* and *BRAF* mutations [168]. These findings are in line with results from the ColoBEAM study, where BEAMing assay technology confirmed high overall tissue and blood concordance for *RAS/BRAF* of 89.3% (Se = 87.5%; Sp = 92.0%) in chemotherapy-naïve patients [171]. These studies highlight that cfDNA extracted from plasma is an attractive surrogate marker to tissue DNA biopsy for *KRAS*, *NRAS* and *BRAF* mutation assessment (Table 2). However, before liquid biopsies can be implemented in clinical practice, it is necessary to not only validate in large prospective clinical trials but also to standardize protocols around blood collection, processing and storage and DNA extraction and quantification methodologies.

### 5.6. RAS and EGFR-ab Therapy

The clinical benefits of EGFR-ab therapies have been demonstrated in patients with mCRC, however, mutations in *RAS* are reportedly linked to resistance [130,174]. Therefore, the identification of *RAS* mutations in tumor tissues to determine patients that are more likely to benefit from anti-EGFR therapies has become standard of care [175]. Moreover, patient tumors that are *RAS^WT^* often develop resistance within several months of initiating therapy, thus limiting the clinical benefit of EGFR-ab therapies [68,69]. The emergence of *RAS* mutations is potentially responsible for acquired resistance to anti-EGFR ab therapy in mCRC [70,176,177]. Diaz and colleagues explored the hypothesis that rare cells with *KRAS* mutations pre-exist at low levels in tumors that are presumed to be *RAS^WT^*. Their retrospective study analyzed longitudinal serum samples from patients with chemo refractory mCRC receiving EGFR-ab therapy [70]. The authors found that 38% of patients with *KRAS^WT^* tumors developed detectable *KRAS* mutations in their sera between five- and six-months following treatment. This study demonstrates the emergence of *KRAS* mutations as a mediator of acquired resistance to EGFR-ab therapy and suggests a mechanism as to why solid tumors develop resistance to targeted therapies [70]. The application of a non-invasive liquid biopsy may be a powerful technology that can be utilized to detect tumor heterogeneity in the form of circulating *RAS* mutations as a biomarker for potential resistance in this subset of patients (Table 2).

### 5.7. PIK3CA Mutations and Adjuvant Aspirin

A number of studies have demonstrated a possible protective effect for regular use of aspirin on colorectal neoplasia [71,72,73,74,75,76,77]. *PIK3CA* mutations are an emerging tumor biomarker that may predict response to adjuvant aspirin treatment. This is currently being prospectively tested in ASCOLT, a large adjuvant study following aspirin use in patients undergoing resection of their CRC [178]. Aspirin suppresses cancer-cell growth and induces apoptosis by blocking the carcinogenic phosphatidylinositide-3-kinases (PI3K) pathway [78,79]. Liao and colleagues were the first to test the hypothesis that post-diagnosis use of aspirin improves survival in mutated *PIK3CA* but not *PIK3CA^WT^* CRC patients. In their prospective two cohort studies, coexistence of *PIK3CA* exon 9 and 20 mutations but not in either exon alone, was associated with significantly worse cancer-specific survival (log-rank *p* = 0.0008; multivariate HR = 3.51; 95% CI, 1.28–9.62] and OS (log-rank *p* = 0.0008; multivariate HR = 2.68; 95% CI, 1.24–5.77) [80] (Table 2). On the contrary, other studies suggest that mutant *PIK3CA* may not be as predictive as first thought [179]. This is highlighted in a study by Kothari and colleagues, who combined data from two large academic institutions and examined the association between regular aspirin use and improved survival in *PIK3CA*-mutated CRC patients. The authors found that regular aspirin use was not associated with improved OS (multivariate HR 0.96; *p* = 0.86) and despite a trend towards improved cancer-specific survival (multivariate HR 0.60; *p* = 0.14), this was not significant [179]. As a result, mutations in *PIK3CA* may serve as a predictive biomarker for adjuvant aspirin therapy, however, further investigations involving prospective randomized studies are warranted.

### 5.8. Biomarkers for Predicting Pathologic Complete Response

The standard of care for locally advanced rectal cancer is neoadjuvant chemoradiation (nCRT) followed by radical surgery. There has been increasing interest in the role for biomarkers in predicting pathologic complete response in rectal cancer patients following nCRT. Six of the most commonly researched biomarkers in this area include p53, EGFR, thymidylate synthase (TYMS), Ki-67, p21 and Bcl-2/bax. The literature evaluating p53, Ki-67 and Bcl-2 does not warrant further exploration due to lack of correlation with biomarker expression and tumor response following nCRT [180,181]. However, there is some evidence to suggest that low expression of TYMS and EGFR is associated with increased tumor regression rates [87,88,89,90,91] and low p21 expression may be associated with improved survival in rectal cancer [92] (Table 2). However, these studies have been performed in small cohorts and conflicting reports exist in the literature [92,182,183,184,185,186]. As a result, biomarkers that predict pathologic complete response have not reached the clinic, most likely due to the lack of prospective studies with reproducible results in large patient cohorts. New technologies and approaches such as improved imaging strategies, microarrays, organoids and searches for circulating molecules may facilitate development in this area. It is important that old and new biomarkers for pathological complete responses be studied in larger prospective trials with consistent staging, treatment and response criteria.

### 5.9. Genetic Alterations

#### 5.9.1. Phosphatase and Tensin Homolog (PTEN)

*PTEN*, a key component of the PI3K/AKT pathway, acts as a tumor suppressor gene and is involved in the regulation of the cell cycle and cellular processes such as proliferation, differentiation and apoptosis [82,83]. *PTEN* deficiency can be caused by several mechanisms including transcriptional, translational and/or post-translational aberration leading to loss of protein expression. This deficiency in *PTEN* is thought to constitutively activate the AKT pathway leading to tumorigenesis [84]. Subsequently, a number of studies have shown that loss of *PTEN* expression correlates with poor survival outcomes and increased metastases to liver and lymph nodes [85,187]. In a meta-analysis of eight randomized control studies, Shen and colleagues investigated the correlation between PTEN expression and cetuximab efficacy in CRC. The authors found that patients with intact *PTEN* protein expression had a better objective response rate to cetuximab-based therapy (RR, 4.75; 95% CI, 2.59–8.72; *p* < 0.001) and better PFS (HR, 0.675; 95% CI, 0.473–0.964; *p* = 0.031). Furthermore, analysis of OS confirmed that loss of *PTEN* was significantly associated with poor clinical outcome (HR, 0.608; 95% CI, 0.411–0.899; *p* = 0.013) [85] (Table 2). However, there are contradictory reports including the study by Eklöf and colleagues who assessed the prognostic role of *PTEN* expression in two patient cohorts. These authors assessed the prognostic significance of *PTEN* expression alone and in combination with *KRAS*, *BRAF* and *PIK3CA*. Their findings concluded that *PTEN* alone, did not add significant prognostic value in both the NSHDS cohort (HR 1.555; 95% CI, 0.859–2.816; *p* = 0.142) and the CRUMS cohort (HR, 0.0870; 95% CI, 0.531–1.426; *p* = 0.581) [86]. Currently, the role for *PTEN* as a prognostic biomarker in CRC remains controversial.

#### 5.9.2. 18q Loss of Heterozygosity

Allelic loss at chromosome 18q, assessed by LOH analysis, results in inactivation of tumor suppressor genes, including *DCC*, *SMAD4*, *SMAD2* and *CABLES1* [93,94,95]. 18q LOH has been inversely associated with MSI, an important molecular classifier in CRC [96]. As a prognostic marker, 18q LOH predicts shorter survival in CRC patients in a number of studies [96,97,98] (Table 2). Popat and Houlston performed a systematic review and meta-analysis on the relationship between chromosome 18q loss of heterozygosity and prognosis in CRC [188]. The pooling of data from 17 studies showed significantly worse OS in patients with chromosome 18q LOH (HR = 2.00; 95% CI, 1.49–2.69) and this was maintained in the adjuvant setting (HR = 1.69; 95% CI, 1.13–2.54). These results are in line with a retrospective study by Jen and colleagues, where the authors assessed 18q status via microsatellite markers and DNA from formalin-fixed, paraffin embedded tumors in patients with resected colon cancer. The findings from their study highlighted in stage II colon cancer patients, allelic loss at chromosome 18q correlated with 54% survival vs. 83% survival in patients without chromosome 18 allelic loss (*p* = 0.0005) [97]. These studies suggest a prognostic role for allelic loss at chromosome 18q for poor prognosis, however, this has been challenged by other reports [99,100]. The findings from these studies indicate that allelic loss of chromosome 18q has the potential to be a prognostic biomarker in CRC, however, further validation in the context of prospective clinical trials utilizing consistent methodologies are warranted.

### 5.10. Immune-Related Biomarkers

The presence of tumor-infiltrating lymphocytes (TILs) within the tumor microenvironment is a recognized prognostic marker in CRC. High densities of infiltrating cytotoxic and memory T-cells in the core and at the invasive margins of the tumor is strongly correlated with survival benefit [101,102,103]. High immune cell densities have been associated with decreased risk of recurrence and tumor dissemination [101,104], as well as better response to chemotherapy [105]. MSI tumors display highly infiltrated CD8+ cytotoxic T lymphocytes and accordingly, have better OS than their MSS counterparts. Due to the requirement of pre-existing anti-tumor T cells, patients with MSI tumors also experience better therapeutic outcomes on immune checkpoint modulators such as anti-PD-L1 [189]. Whilst TILs are a strong indicator of response to immunotherapy, there is conflicting evidence over the predictive value of PD-1 and PD-L1 [190,191,192,193]. Similarly, the prognostic significance of PD-L1 in CRC is limited and remains controversial. Li and colleagues performed a systematic review and meta-analysis on 10 studies to determine the prognostic significance of PD-L1 expression in CRC patients [194]. The authors found tumor PD-L1 expression was significantly associated with poor OS (HR, 1.50; 95% CI, 1.05–2.13; *p* = 0.03) and shorter DFS (HR, 2.57; 95% CI, 1.140–4.75; *p* = 0.002) [194]. These findings highlight that high level of PD-L1 expression might be a biomarker for poor prognosis in CRC patients. In addition, some studies indicate that patients with PD-L1-positive tumors are more likely to respond to anti-PD-1/PD-L1 blockade therapy, additional parameters would be required to more accurately distinguish predicted responders from non-responders [190]. Further investigation is required to validate the use of immune-related biomarkers in sizeable well-designed cohort studies.

### 5.11. Apoptosis-Related Biomarkers

Apoptotic pathways are frequently altered in tumor progression and subsequently proteins associated with this pathway may have potential as prognostic biomarkers [106]. These apoptosis-related biomarkers include B-cell lymphoma-2 (Bcl-2) like proteins, Bax family, BH3 proteins, caspases and inhibitors or apoptosis proteins.

The most frequently examined apoptotic protein for potential clinical use is Bcl-2 which has been examined as a prognostic biomarker in a number of cancers, including ovarian, lung and gastric cancer [107,108,109]. Aberrant expression of Bcl-2 has been implicated in CRC [195,196]. A number of studies suggest expression of BcL-2 correlates with clinicopathologic parameters and better prognosis [197,198,199,200]. Conversely, others suggest that Bcl-2 is a poor prognostic marker [201,202,203,204,205,206]. Huang and colleagues performed a meta-analysis on 40 CRC articles involving 7,658 patients and found high Bcl-2 expression correlated with favorable OS (pooled HR, 0.69; 95% CI, 0.55–0.87; *p* = 0.002) and better DFS/RFS (pooled HR, 0.65; 95% CI, 0.50–0.85; *p* = 0.001). In their sub analyses, the authors suggest that Bcl-2 was a favorable prognostic factor in subgroups with greater than 100 patients and in those with European and American countries of origin. These findings suggest that Bcl-2 may be a useful prognostic marker although further research, including the impact of ethnicity, is required before being incorporated into clinical practice.

## 6. Future Directions

There are many exciting developments in the area of CRC biomarker discovery. The discovery of novel means for investigating biomarkers, including recent characterization of CMS, liquid biopsies, patient-derived xenografts and organoids, open opportunities that may contribute towards and enhance biomarker discovery and will be discussed below. It is worth noting that the gold standard for biomarker development has still not been decided, with further work required to prospectively validate its use. Further research is needed to make this standard clinically viable, especially in the areas of reproducibility, quantification and commercially expandable [70].

Current treatment strategies for CRC patients with advanced disease are evolving from standard, staging-based therapies to mechanism-based therapeutics, guided by the molecular profile of the individuals’ tumor rather than tumor tissue type or anatomical location. The new classification of CRC has opened the door to personalize treatment based on a CMS subtype [123]. At present, the current methodologies in characterizing the CMS are not easily translated into clinical practice, such as obtaining high quality genome-wide transcriptome data from formalin-fixed paraffin embedded tissue. However, emerging research has highlighted IHC based classifiers for the CMS with five markers (CDX2, FRMD6, HTR2B, ZEB1 and KER) achieving 87% concordance with the gold standard transcriptome-based classification [124]. This has potential for further refinement and integration into future routine clinical care. In addition, the incorporation of tissue microarrays (TMAs) with IHC analysis of CMS may provide a high throughput, rapid and cost-effective approach to examining CMS. However, further validation studies are required.

The role for patient-derived xenografts are increasing within the area of biomarker discovery. This experimental model of cancer involves transplantation of a patient’s tumor cells into immunodeficient mice [207]. These models are biologically stable and accurately reflect the patients’ tumors with regard to histopathology, gene expression, genetic mutations, inflammation and therapeutic response [208] allowing the tumors to mimic the microenvironment of the patient’s tumor where there is cell-cell interaction, unlike cell lines. This experimental model also allows for the administration of anti-cancer drugs or antibodies to the mouse to test for efficacy towards a particular cancer [209]. The use of patient derived xenografts in biomarker discovery is, however, limited by several factors including the replacement of human stromal components (i.e., cancer-associated fibroblasts, immune/inflammatory cells and endothelial cells) by murine elements, the lack of a functional immune system and the lack of interactions between the immune system and human stromal cells. As a result, improvements in the development of humanized models, such as the patient-derived xenograft model, that incorporate cancer-stromal interactions will need to be developed to enhance biomarker discovery.

Another innovative tool for biomarker discovery are patient-derived tumor organoids, a three-dimensional in vitro model that has considerable potential for use as an ex vivo platform to predict and personalize treatment outcomes for patients [210,211]. Tumor organoids maintain the biological fidelity of the primary lesion from which they are derived in terms of gene expression and genetic stability. These features allow tumor organoids to recapitulate the cellular heterogeneity of patient tumors at a greater level than traditional models [212,213,214,215,216,217,218]. Tumor organoid technology is recognized for generating reproducible, high quality drug sensitivity data and can be highly effective in identifying and evaluating biomarkers that underpin drug sensitivity [58]. There is also significant potential for discovery of novel combinatorial drug treatments and repurposing of drugs with previously unknown therapeutic benefit with organoid technology amenable to high-throughput screening [213,219]. However, large scale, multi-centered studies are required to validate this approach for prediction of patient responses.

## 7. Conclusions

The future of personalized medicine is dependent on the incorporation of robust and validated biomarkers. Developing personalized treatment strategies by conducting research and prospective trials that interrogate the processes involved in cancer will lead to reduced patient morbidity and mortality. The ultimate goal is to understand and identify patients that would benefit from surgery or current therapeutics by assessing the risk-benefit of different cancer therapies based on the characteristics of the patient’s individual tumor profile. Continued research into the area of biomarkers and personalized medicine will bring greater insights into the genetic and molecular defects that underpin CRC and lead to improved patient outcomes.

## Figures and Tables

**Table 1:** Current clinical biomarkers and their clinical utility.

Clinical Biomarkers	Role	Clinical Utility	References
dMMR	Diagnosis/Therapy choice	Widespread use. Testing for loss of DNA MMR proteins (MLH1, MSH2, MSH6, PMS2) is typical of Lynch Syndrome/HPNCC. Used to indicate contraindication for the use of fluoropyrimidine chemotherapy.	[3,4,5,6,7,8,9]
MSI	Diagnosis/Prognosis/Therapy choice	Widespread use. MSI tumors have a better prognosis. May suggest possible resistance to fluoropyrimidine chemotherapy. MSI-H tumors are highly responsive to immunotherapy.	[10,11]
*KRAS*	Prognosis/Therapy choice	*KRAS* mutations indicate unresponsiveness to EGFR-ab therapies.	[12,13,14,15,16,17,18,19,20,21,22]
*BRAF*	Prognosis	*BRAF* mutations indicate a decreased survival rate.	[23,24]
CEA	Diagnosis/Prognosis	Widespread use. A rising CEA post-surgery often correlates with relapse.	[25,26,27,28]
UGT1A1*28	Therapy choice	UGT1A1*28 polymorphism is associated with irinotecan toxicity.	[29]
DPD	Therapy choice	DPD deficiency may lead to life threatening toxicity of fluoropyrimidine chemotherapy.	[30]
*APC*	Diagnosis	*APC* mutations are common in the autosomal dominant FAP syndrome, with confirmation of FAP by colonoscopy.	[31,32,33]
*SMAD4*, *BMPR1A*	Diagnosis	40% of Juvenile polyposis syndrome (JPS) cases have *SMAD4* and *BMPR1A* gene mutations.	[34]

Abbreviations: *APC*; adenomatous polyposis coli gene, *BMPR1A*; bone morphogenetic protein receptor, type IA, CEA; Carcinoembryonic antigen, dMMR; deficient mismatch repair, DPD; dihydropyrimidine dehydrogenase, EGFR-ab; Epidermal growth factor receptor antibody, FAP; Familial adenomatous polyposis, HNPCC; Hereditary nonpolyposis colorectal cancer, JPS; Juvenile polyposis syndrome, MSI; Microsatellite instability. MSI-H; Microsatellite instability high.

**Table 2:** Potential emerging biomarkers and their clinical utility.

Emerging Biomarkers	Potential Role	Potential Clinical Utility	References
CMS	Therapy Choice	CMS4 tumors may predict whether a patient responds to irinotecan. CMS2 and possibly CMS3, tumors benefit from addition of bevacizumab to first line capecitabine-based chemotherapy in mCRC.	[35,36]
CIMP	Prognosis	Tumors with hypermethylation in the promoter regions of tumor suppressing genes with MSI and *BRAF* mutations have a good prognosis. Tumors that are CIMP positive, MSI negative and *BRAF* mutated have poor prognosis.	[37,38,39,40,41,42,43]
DNA aneuploidy	Prognosis	DNA aneuploidy is linked to poor prognosis in Stage II-III CRC.	[44,45,46,47,48]
Stem cell markers	Prognosis	‘Stem cell signature’ on cancer cells is associated with more aggressive tumors and predicts disease relapse.	[49,50,51,52,53,54,55,56,57,58]
ctDNA and cfDNA	Prognosis	ctDNA in blood tests could be used to predict whether a patient would relapse following surgical resection. cfDNA in blood tests could predict shorter overall survival and inferior recurrence free survival.	[59,60,61,62,63,64,65,66,67]
*RAS*	Prognosis/Therapy choice	Testing for *RAS* in patient blood may predict whether a patient will be resistant to EGFR-ab therapies.	[68,69,70]
*PIK3CA* mutations	Prognosis/Therapy choice	Mutations in *PIK3CA* may be predictive for the effectiveness of adjuvant aspirin therapies.	[71,72,73,74,75,76,77,78,79,80,81]
*PTEN*	Prognosis	Loss of *PTEN* in tumors is associated with shorter progression free survival.	[82,83,84,85,86]
*TYMS, EGFR* and *p21*	Prognosis/Therapy choice	Low expression of TYMS and EGFR is associated with increased tumor regression rates. Low p21 expression may be associated with improved survival in rectal cancer.	[87,88,89,90,91,92]
18q loss of heterozygosity (LOH)	Prognosis	18q LOH predicts lower overall survival in CRC.	[93,94,95,96,97,98,99,100]
TIL	Prognosis	High density of TILs is correlated with better survival.	[101,102,103,104,105]
Bcl-2	Prognosis	Loss of Bcl-2 expression is correlated with tumor recurrence.	[106,107,108,109,110,111,112]

Abbreviations: CMS; consensus molecular subtype, CIN; chromosome instability, CRC; colorectal cancer, cfDNA; cell-free DNA, ctDNA; circulating tumor DNA, EGFR; Epidermal growth factor receptor, EGFR-ab; Epidermal growth factor receptor antibody, LOH; loss of heterozygosity, MSI; Microsatellite instability, nCRT; neoadjuvant chemoradiotherapy, *PIK3CA*; Phosphatidylinositol-4, 5-Bisphosphate 3-Kinase Catalytic Subunit Alpha, *PTEN*; Phosphatase and tensin homolog, TIL; tumor infiltrating lymphocyte, TYMS; thymidylate synthase.
